# The distribution of fitness effects of plasmid pOXA-48 in clinical enterobacteria

**DOI:** 10.1099/mic.0.001369

**Published:** 2023-07-28

**Authors:** Ariadna Fernández-Calvet, Laura Toribio-Celestino, Aida Alonso-del Valle, Jorge Sastre-Dominguez, Paula Valdes-Chiara, Alvaro San Millan, Javier DelaFuente

**Affiliations:** ^1^​ Centro Nacional de Biotecnología (CNB-CSIC), Madrid, Spain; ^2^​ Centro de Investigación Biológica en Red de Epidemiología y Salud Pública (CIBERESP), Instituto de Salud Carlos III, Madrid, Spain

**Keywords:** antimicrobial resistance, carbapenemase, conjugation, enterobacteria, pOXA-48, plasmids, DFE, Plasmid cost

## Abstract

Antimicrobial resistance (AMR) in bacteria is a major public health problem. The main route for AMR acquisition in clinically important bacteria is the horizontal transfer of plasmids carrying resistance genes. AMR plasmids allow bacteria to survive antibiotics, but they also entail physiological alterations in the host cell. Multiple studies over the last few years have indicated that these alterations can translate into a fitness cost when antibiotics are absent. However, due to technical limitations, most of these studies are based on analysing new associations between plasmids and bacteria generated *in vitro*, and we know very little about the effects of plasmids in their native bacterial hosts. In this study, we used a CRISPR-Cas9-tool to selectively cure plasmids from clinical enterobacteria to overcome this limitation. Using this approach, we were able to study the fitness effects of the carbapenem resistance plasmid pOXA-48 in 35 pOXA-48-carrying isolates recovered from hospitalized patients. Our results revealed that pOXA-48 produces variable effects across the collection of wild-type enterobacterial strains naturally carrying the plasmid, ranging from fitness costs to fitness benefits. Importantly, the plasmid was only associated with a significant fitness reduction in four out of 35 clones, and produced no significant changes in fitness in the great majority of isolates. Our results suggest that plasmids produce neutral fitness effects in most native bacterial hosts, helping to explain the great prevalence of plasmids in natural microbial communities.

## Data availability

The sequencing data supporting the findings of this study are available at the National Center for Biotechnology Information Database with accession number PRJNA962867. The closed genomes of the six wild-type strains can be accessed from PRJNA626430. The raw experimental data obtained in this study are available in Table S1. The remaining R-GNOSIS sequences can be found in León-Sampedro *et al*. [1].

## Code availability

The code generated during the study can be found at https://github.com/LaboraTORIbio/CRISPR_cured_pOXA-48

## Introduction

Plasmids are extrachromosomal genetic elements able to spread horizontally between bacteria. Plasmids contain genes responsible for their own replication, partition and transfer, as well as accessory genes that can provide a selective advantage to the host bacterium. Crucially, plasmids fuel bacterial evolution by disseminating accessory genes across populations [2, 3]. Antimicrobial resistance (AMR) is one of the most illustrative examples of the ability of plasmids to promote bacterial adaptation [4]. AMR is a major public health issue [5], and plasmids are the most important vehicle for the dissemination of AMR genes across bacterial pathogens [6]. AMR is particularly concerning in clinical settings, where nosocomial pathogens carrying AMR plasmids represent a great threat to hospitalized patients [5, 7].

Despite the selective advantage that plasmids may provide, they also produce physiological alterations in the bacterial host that can translate into a fitness cost [8–14]. These plasmid-associated costs raise the paradox of how plasmids are maintained in bacterial populations [15]. Specifically, in the absence of selective pressure for plasmid-encoded traits, purifying selection should remove plasmid-carrying bacteria, while in the presence of selection for plasmid-encoded genes, these loci would eventually be captured by the chromosome, making the plasmid redundant. Different solutions have been proposed for this plasmid paradox (reviewed in [16]). A simple solution is that plasmid fitness effects differ across different bacterial hosts, and some strains experience no costs at all. We recently showed that this is indeed the case for pOXA-48 [17], a conjugative plasmid conferring carbapenem resistance, which is widely distributed across nosocomial enterobacteria worldwide [18]. In a previous study, we introduced pOXA-48 into a collection of ‘ecologically compatible’ clinical enterobacteria [17]. We defined these isolates as ecologically compatible with the plasmid because they were recovered from hospitalized patients who although not colonized by pOXA-48-carrying enterobacteria, coexisted in hospital wards with patients colonized by pOXA-48-carrying enterobacteria. The plasmid produced a 3 % average fitness reduction in this collection; however, we observed a wide distribution of fitness effects across strains, including fitness advantages in some of them.

One general limitation in the study of plasmid fitness effects is that most of the works to date have studied the effects of plasmids that are introduced *de novo* in naive bacterial strains. Therefore, there is little information about the fitness effects of plasmids in the natural bacterial hosts that actually carry them [19, 20]. The reason underlying this limitation is the lack of proper methods for selectively eliminating (curing) plasmids from wild-type bacteria without producing further genetic alterations. In this study, we used a CRISPR-Cas9-based method that we recently developed [21] to specifically cure a collection of pOXA-48-carrying enterobacteria that were recovered from hospitalized patients. With this method, we produced 35 isogenic pairs of wild-type and pOXA-48-free strains that allowed us to precisely estimate pOXA-48 fitness effects.

## Methods

### Strains, media and culture conditions

**Fig. 1.**
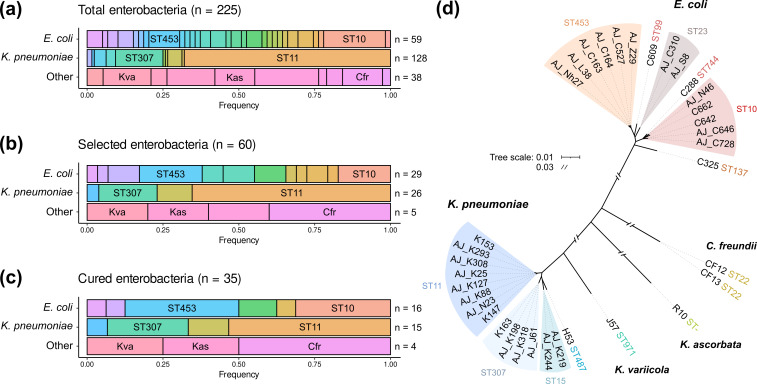
Bacterial collection used in this study. Summary of pOXA-48-carrying strains present in the entire collection and the subset used for this study. (**a**) Distribution of multilocus sequence types (STs) for *E. coli *, *Klebsiella pneumoniae* and other species in the collection of wild-type pOXA-48-carrying enterobacteria. (**b**) Representative subsample of the collection selected for this study. (**c**) Strains successfully cured from pOXA-48. Colours indicate different STs (upper and central rows) or different species (lower row). The most representative STs in *E. coli* and *K. pneumoniae* are indicated. Kva: *Klebsiella variicola *; Cfr: *Citrobacter freundii *; Kas: *Kluyvera ascorbata *. (**d**) Unrooted phylogeny reconstructed with the genomic sequences of the 35 strains cured from pOXA-48. Branch length represents mash distances between whole-genome assemblies. Scale breaks were introduced for long branches. The STs are also indicated.

Bacterial clones carrying pOXA-48-like plasmids included in this study were initially isolated and characterized as part of a surveillance screening for detecting extended-spectrum β-lactamases/carbapenemases in hospitalized patients in the Hospital Universitario Ramon y Cajal (Madrid, Spain) (R-GNOSIS-FP7-HEALTH-F3-2011-282512; http://r-gnosis.eu/). All clones included in this study were recovered as part of a project approved by the Hospital Universitario Ramon y Cajal Ethics Committee (ref. no. 251/13). Further details of the R-GNOSIS isolation protocol and sample characterization can be found in the literature [1, 21, 22]. To study the distribution of plasmid-associated fitness effects in wild-type strains naturally carrying pOXA-48-like plasmids, a representative subset of 60 clones which included the most common sequence types (STs) in the collection (determined by multilocus sequence types) was randomly generated (Table S2, available in the online version of this article). Note that the most predominant STs in the collection were maintained in the final selection (*n*=35), but not the final proportions of each ST from the initial collection (*n*=225), as shown in Fig. 1(a–c) and Table S2. For antimicrobial susceptibility testing, Mueller Hinton II broth (MH; Oxoid) was used. Lennox lysogeny broth (LB) was used for plasmid curing, growth curves and competition assays. Chromogenic agar (HiCrome UTI Agar; HIMEDIA) was used for plasmid curing. When indicated, solid media were obtained by supplementing each medium with 15 g l^–1^ agar (CONDA). Amoxicillin-clavulanic acid (Sandoz), ertapenem, chloramphenicol, apramycin and kanamycin (Merck) were used in this study. pLC10 purification was performed with plasmid EasyPure (Macherey-Nagel).

### pOXA-48 curing

The synthetic pLC10 plasmid carrying the CRISPR-Cas9 machinery was used to cure the native pOXA-48 plasmid from the wild-type enterobacteria. Two different versions of pLC10 were used depending on the resistance profile (Table S2) of each bacterium: pLC10-Kan (kanamycin-resistant) and pLC10-Apra (apramycin-resistant). A full description of the plasmid can be found in Fig. S1 and in DelaFuente *et al*. [21]. All primers used in this study are listed in Table S3. Importantly, pLC10 codes for a thermosensitive replication initiation protein (pSC101-based) which is not functional at 37 °C. Cas9 protein expression is under the control of a P*_tet_* promoter which is inducible with anhydrotetracycline (ATC). Two different single-guide RNAs (sgRNAs) were used targeting two different pOXA-48 genes (*pemK* or *bla*_OXA-48_) and differences in curing efficiency depending on the sgRNA were not detected. sgRNA expression is under the control of a P*_lac_* promoter, inducible with IPTG. Each sgRNA was introduced by Golden Gate assembly (New England Biolabs). Primers used for cloning each sgRNA were: (i) for the *pemK* region, Fw 5′-CACAGTTGTGCCCGTGACCAGCGG-3′ and Rev 5′-AAACCCGCTGGTCACGGGCACAAC-3′; and (ii) for *bla*_OXA-48_, Fw 5′-CACATGGCTTGTTTGACAATACGC-3′ and Rev 5′-AAACGCGTATTGTCAAACAAGCCA-3′. Later, pOXA-48-carrying strains were made competent: we cultured each clone overnight at 37 °C with continuous agitation (250 r.p.m.; MaxQ 8000; Thermo Fisher Scientific). Then, overnight cultures were diluted 1 : 100 in LB and after 2.5 h of culturing in the same conditions as the day before, cells were harvested by centrifugation (3000 ***g***). Then, bacterial cells were washed with distilled ice-cold water (Invitrogen) several times. Later, pLC10 was introduced into bacterial cells by electroporation using 0.1 cm cuvettes/1.8 kV pulse following the manufacturer’s recommendations (MicroPulser Electroporator; Bio-Rad Laboratories). Transformants were selected on LB agar plates supplemented with kanamycin 250–512 µg ml^−1^ or apramycin 30–50 µg ml^−1^, depending on the plasmid version. pLC10 presence was determined by PCR (Fw 5′-CTCGGTAGTGGGATACGACGA-3′ and Rev 5′-CACTGAAAGCACAGCGGCTG-3′; amplicon size 859 bp). Then, CRISPR-Cas9 machinery was induced by resuspending transformant biomass in 500 µl of LB supplemented with kanamycin or apramycin and 0.3 µg ml^−1^ of ATC to induce Cas9 expression, and 0.08 mM IPTG to induce sgRNA expression. After 3 h of incubation with continuous agitation (250 r.p.m.; MaxQ 8000; Thermo Fisher Scientific), suspensions were streaked and incubated overnight at 37 °C on LB agar to eliminate pLC10. The next day, single colonies were serially streaked on LB agar supplemented with ertapenem (ERT, 0.5 µg ml^−1^), LB agar supplemented with kanamycin (250–512µg ml^−1^) or apramycin (30–50 µg ml^−1^), and antibiotic-free LB agar. Plates were incubated overnight at 37 °C and only colonies able to grow in LB, but not on the other two plates, were selected. Then, pLC10 absence was confirmed by PCR (as described above) and pOXA-48 absence was determined by PCR using primers targeting two specific different pOXA-48 conserved regions: (i) the *bla*_OXA-48_ gene (Fw 5′-TTGGTGGCATCGATTATCGG-3′, Rev 5′-GAGCACTTCTTTTGTGATGGC-3′; amplicon size 744 bp) and (ii) *repC* gene (Fw 5′-CGGAACCGACATGTGCCTACT-3′ and Rev 5′-GAACTCCGGCGAAAGACCTTC-3′; amplicon size 852 bp). Confirmed pOXA-48 and pLC10-free colonies were resuspended in LB supplemented with 13 % glycerol and stored at −70 °C. Additionally, each pOXA-48-carrying/pOXA-48-free bacterial pair was validated with chromogenic medium and antimicrobial susceptibility testing, to determine that the phenotypic profile was consistent with being the same clone with or without pOXA-48 (Table S4). Single colony isolates derived from the curing process were stored. Then, pOXA-48-carrying and pOXA-48-free bacteria were used to inoculate 2 ml LB starter cultures, which were incubated overnight at 37 °C with continuous agitation (250 r.p.m.; MaxQ 8000; Thermo Fisher Scientific). Each culture was diluted 1 : 40 in 2 ml of 0.9 % NaCl (~10^7^ c.f.u. ml^–1^) and swabs were used to spread the cell suspension on the MH plates. Antibiotic discs (Bio-Rad) were placed on the plates. Plates were incubated at 37 °C for 24 h and the diameter of the growth inhibition halos were measured. Antibiotic discs tested were: azythromycin 15 µg (AZM15), tetracycline 30 µg (TET30), cefotaxime 30 µg (CTX30), streptomycin 10 µg (SMN10), amoxicillin+clavulanic acid 20/10 µg (AMC30), rifampicin 5 µg (RIF5), fosfomycin 200 µg (FOS200), chloramphenicol 30 µg (CHL30) and meropenem 10 µg (MEM10).

### Bacterial growth curves

Bacterial cultures were inoculated from freezer stocks in 2 ml of LB and incubated overnight at 37 °C with continuous shaking (250 r.p.m.; MaxQ 8000; Thermo Fisher Scientific). Then, each culture was diluted 1 : 1000 into fresh LB and was used to fill flat-bottom 96-well plates with 200 µl per well (Thermo Fisher Scientific). Eight replicates from each genotype were included. Then, bacterial cultures were incubated for 24 h at 37 °C in a plate reader (Synergy HTX Multi-Mode Reader; BioTek Instruments). During incubation, optical densities (OD_600_) were measured after 15 s of orbital agitation (282 c.p.m., 3 mm) every 10 min. After incubation, and to discard the potential loss of pOXA-48 during the growth cycles, two replicates of each genotype were serially diluted in 0.9 % NaCl and plated on LB agar plates supplemented with and without amoxicillin+clavulanic acid (250/50 µg ml^−1^; Normon). Agar plates were incubated overnight at 37 °C and counts (c.f.u. ml^–1^) were estimated. The growth curve data were analysed using Rstudio 2022.12.0+353 (R v4.2.2) and *flux* v.0.3.0.1 was used to determine the area under the curve (AUC).

### Relative fitness determination by competition assays

To calculate the relative fitness of pOXA-48-carrying isolates compared to their pOXA-48-free counterparts, competition assays were performed by flow cytometry (CytoFLEX Platform; Beckman Coulter) as previously described [17]. Cytometer parameters were: 50 µl min^−1^ flow rate, 22 µm core size and 10 000 events per well. *Escherichia coli* J53 [23] (a sodium azide-resistant mutant of *E. coli* K-12) carrying the pBGC plasmid (accession number MT702881, Fig. S1C) was used as a common competitor in all competition assays. pBGC is a non-mobilizable plasmid that contains the *gfp* gene under the control of the P*_BAD_* promoter. Hence, GFP production is induced by the presence of arabinose. Two sets of competitions were performed for each isolate: pOXA-48-free vs. *E. coli* J53/pBGC, and pOXA-48-carrying vs. *E. coli* J53/pBGC with six replicates per competition. Initial pre-cultures were inoculated from freezer stocks in 200 µl of LB and incubated overnight with continuous shaking at 225 r.p.m. at 37 °C in 96-well plates (Thermo Fisher Scientific). On the next day, bacterial cultures were mixed 1 : 1 and diluted 10 000-fold in fresh LB. Then, mixtures were competed for 22 h in LB at 37 °C and 225 r.p.m. To determine the initial proportions, initial mixes were diluted 2000-fold in 200 µl of 0.9 % NaCl with 0.1 % l-arabinose (Sigma-Aldrich), and incubated at 37 °C at 225 r.p.m. for 1.5 hours to induce GFP expression before performing flow cytometry determinations. After 22 h of incubation, final proportions were again determined after a 2000-fold dilution of the cultures under the same conditions as before. The fitness of each strain relative to the same common competitor *– E. coli* J53/pBGC – was determined using equation 1:

(1) $$w=\frac{\ln\left(\frac{Nf}{Ni}\right)}{ln(\frac{Nf,pBGC}{Ni,pBGC})}$$

where *w* is the relative fitness of the pOXA-48-carrying or pOXA-48-free isolates compared to the *E. coli* J53/pBGC, *Ni* and *Nf* are the number of cells of the pBGC-free clone at the beginning and end of the competition, and *Ni*,*pBGC* and *Nf*,*pBGC* are the number of cells of the pBGC-carrying *E. coli* J53 at the beginning and end of the competition, respectively. The fitness of the pOXA-48-carrying parental isolates relative to the pOXA-48-free cured isolates (*w_p_*) was calculated using the average result of six independent replicates of each competition and using equation 2:

(2) $$w_{p}=\frac{w_{poxa-48(+)}}{w_{poxa-48(-)}}$$

where *w*_*poxa*-48(+)_ corresponds to the relative fitness of the pOXA-48-carrying clone and *w*_*poxa*-48(-)_ to the relative fitness of the pOXA-48-free clone, both compared to the common *E. coli* J53/pBGC competitor. The error propagation method was used to calculate the standard error of the resulting value. Additionally, *gfp* induction was monitored in each competition by growing individual *E. coli* J53/pBGC cultures. Potential plasmid loss during the competition was discarded by controlling for plasmid loss during growth (Fig. S3). Importantly, controls to discard for potential conjugative transfer of pOXA-48 during competitions were performed by plating the final time points of two replicates of each competition assay on ertapenem (0.5 µg ml^−1^), chloramphenicol (200 µg ml^−1^) and sodium azide (100 µg ml^−1^). No transconjugants were detected, probably due to the small initial population size of the competing populations and the strong shaking regime.

### Genomic DNA extraction and sequencing

Genomic DNA (gDNA) was extracted using the Wizard gDNA purification kit (Promega). From the 35 wild-type strains that were cured from pOXA-48, a subset of 22 strains was sequenced at SeqCoast Genomics (https://seqcoast.com/), using the NextSeq 2000 platform (coverage > 60×). Sequencing data are available under the BioProject PRJNA962867. For the remaining 13 wild-type strains, we used the Illumina data generated previously [1] (BioProject PRJNA626430). The genomes of six wild-type strains were also sequenced with long reads to generate closed references, either with Nanopore technology at the Microbial Genome Sequencing Center (MiGS) or with PacBio RSII at The Norwegian Sequencing Centre (see Table S2). Long-read data and closed assemblies were submitted to SRA and GenBank under the BioProject PRJNA626430. To control for putative curing-derived off-target mutations, a subset of nine cured strains was sequenced at the Wellcome Trust Centre for Human Genetics (Oxford, UK), using the Illumina HiSeq4000 platform, or the MiGS, using the NextSeq 2000 platform (Table S2; data available at BioProject PRJNA962867). The closed sequences of three wild-type strains were retrieved from PRJNA838107 [21] (Table S2).

### Bioinformatic analyses

A detailed description of the bioinformatics methods is provided at https://github.com/LaboraTORIbio/CRISPR_cured_pOXA-48. Illumina reads were trimmed with Trim Galore v0.6.4 (options --quality 20 --length 50 --illumina --paired, https://github.com/FelixKrueger/TrimGalore) or Trimmomatic [24] v0.39, using the maximum information quality filtering algorithm (MAXINFO:50 : 0.8) and removing adapters. Hybrid assemblies of the wild-type strains C325, CF12, CF13, H53, J57 and K147 were obtained with Unicycler [25] v0.4.9. *De novo* genome assemblies were generated with SPAdes [26] v3.15.2 (options --isolate --cov-cutoff auto). Assembly quality was assessed with Bandage [27] v0.8.1 or QUAST [28] v5.0.2. Complete genomes were annotated with PGAP [29] v2021-07-01.build5508 and draft assemblies with Prokka [30] v1.14.6. Multilocus STs were assigned with MLST v2.21.0 (https://github.com/tseemann/mlst).

Phylogenetic trees based on mash distance were reconstructed with mashtree [31] v1.2.0 using the whole-genome assemblies and a bootstrap of 100.

Variant calling was performed with breseq [32] v0.35.6 or v0.35.7. First, to discard false positive calls due to misassemblies, the Illumina reads of the wild-type strains were mapped to their respective closed genomes. Then, the Illumina reads of the cured strains were mapped to their corresponding wild-type reference to identify mutations in cured strains. To further confirm or discard confusing mutations, the Illumina reads of the wild-type strains were mapped to the draft genomes of the cured strains. All mutations reported by breseq – SNPs, missing coverage (deletions) and new junction (genomic rearrangements) evidence – were analysed.

The identified mutations were further characterized with different methods depending on their nature. Functional effects of missense variants were predicted with the SNAP2 [33] webserver (https://rostlab.org/services/snap2web/), where scores <0 indicate neutral effects on protein function. Synonymous variants were not investigated since all would be predicted as neutral. PSIPRED [34] v4.0 was used to predict changes in protein secondary structure caused by frameshift variants, and domain information was obtained from InterProScan [35] 5. The nucleotide sequences of the affected intergenic regions were scanned for promoters using phiSITE’s PromoterHunter [36, 37]. The nucleotide sequences of the mutated genes and intergenic regions were submitted to a BLASTn [37] search against the NCBI database [nr/nt collection, restricted to Enterobacteria (taxid:543)] to determine if the mutations are present in other enterobacteria.

To determine whether the identified variants could be due to off-target cuts of the CRISPR-Cas9 system, the nucleotide sequences of the guides sgOXA48 and sgPEMK were aligned to a 40 bp subregion enclosing the mutations using EMBOSS Needle [38] v6.6.0.0. This tool finds the best global alignment between two sequences. Therefore, if mutations were produced by the CRISPR-Cas9 system, Needle would show the sgRNA alignment that most probably caused the off-target cleavage.

The selective elimination of plasmid pOXA-48 from all cured strains was confirmed by inspecting the missing coverage evidence reported by breseq and by identifying plasmid replicons with ABRicate v1.0.1 (https://github.com/tseemann/abricate), using the PlasmidFinder database.

### Statistical analysis

Statistical analyses were performed in RStudio (R v4.2.2) with R base and the packages *tidyverse*, *outliers*, *ggplot2* and *ggpubr*. To test for homoscedasticity and normality, Bartlett and Shapiro–Wilk tests were performed. Then, according to each data structure, parametric and non-parametric tests were performed (see Results for each test). To compare the distributions of fitness effects between our collection and Alonso-del Valle *et al*.’s 2021 collection, different tests were performed. The data in our collection did not follow a normal distribution (Shapiro–Wilk test, *P*=7.36×10^−6^), since strain AJ_N46 is an outlier (Grubbs test, *P*=1.07×10^−6^), although both data distributions did not differ according to a Kolmogorov–Smirnov test (*D*=0.17, *P*=0.56). On removing the outlier, the data distribution was normal (Shapiro–Wilk test, *P*=0.095). In addition, the means of both distributions were not different as reported by a two-sample *t*-test (*t*=0.23, d.f.=77.67, *P*=0.83) and Wilcoxon rank sum test (*W*=923, *P*=0.6715).

## Results

### Curing pOXA-48 from wild-type clinical enterobacteria

**Fig. 2.**
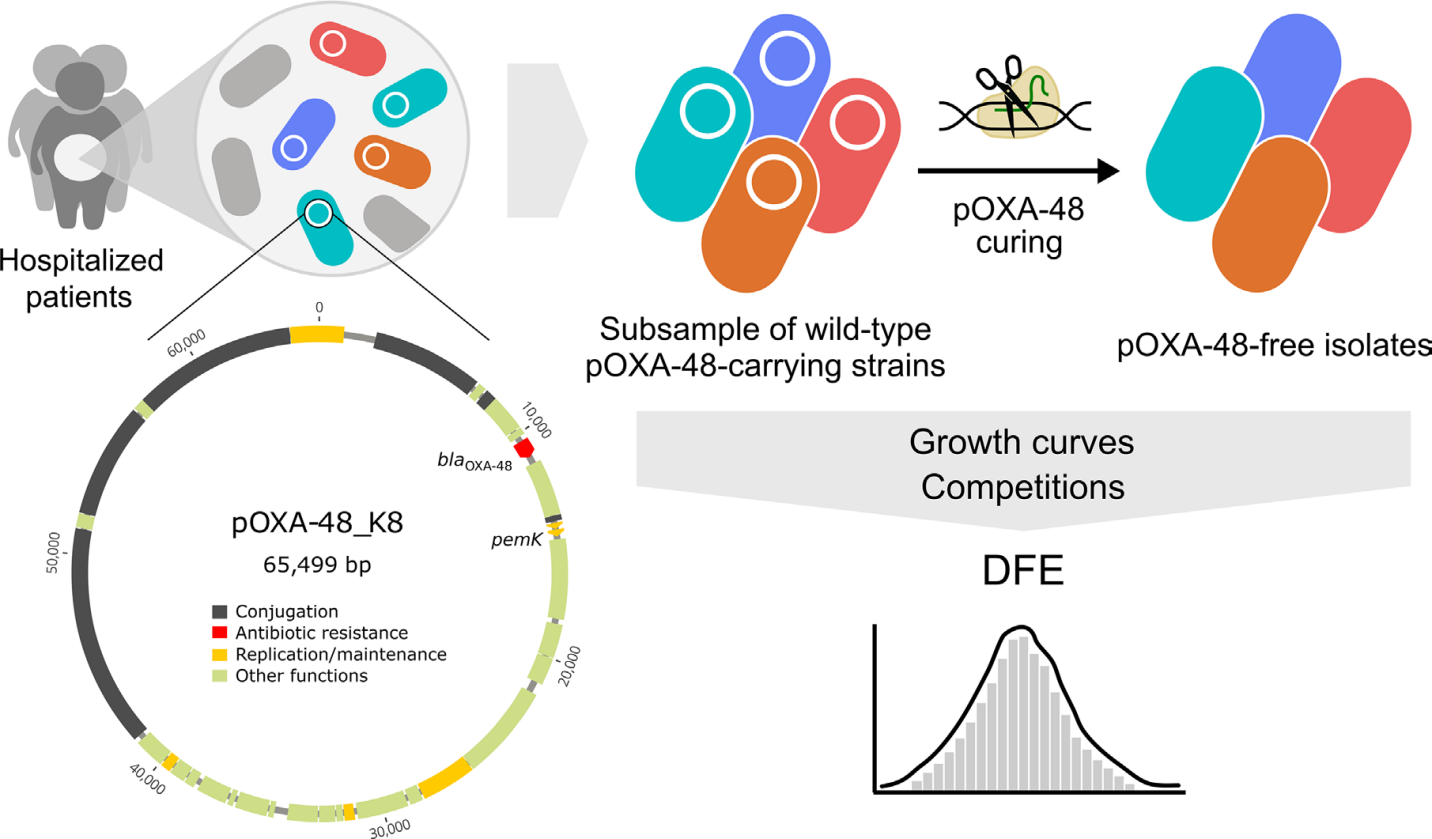
Experimental design. A representative subsample of wild-type pOXA-48-carrying enterobacteria (*n*=60) was selected from a collection isolated from the gut microbiota of hospitalized patients (*n*=225). Plasmid pOXA-48 was eliminated (cured) from the wild-type strains using a CRISPR-Cas9-based method (see Methods). Growth curves and competition experiments were performed for all the pOXA-48-carrying and pOXA-48-free clones. We used the relative fitness data obtained from the competition assays to determine the distribution of fitness effects (DFE) of pOXA-48 in its natural hosts.

In this study, we aimed to determine the distribution of pOXA-48 fitness effects in wild-type strains naturally carrying this plasmid (the outline of this study is presented in Fig. 2). We recently performed a genomic characterization of a large collection of 225 pOXA-48-carrying enterobacteria recovered from patients in a large hospital in Madrid, Spain [1] (Fig. 1a; Table S2). In the present study, we randomly selected a subset of 60 strains from that collection (Fig. 1b; Table S2). We were able to transform our curing vector (pLC10) in 44 of them, and we successfully cured 35 clones belonging to five different species (Fig. 1c, d; Table S2); see Methods and Fig. S1 for details of the curing protocol.

### Genomic validation of the plasmid curing system

To validate the use of our CRISPR-Cas9 system to selectively cure pOXA-48, we sequenced and analysed in detail the genomes of nine cured strains. These included two *E. coli* (C288 and C325), two *Citrobacter freundii* (CF12 and CF13), one *Klebsiella variicola* (J57) and four *Klebsiella pneumoniae* (H53, K147, K153 and K163). The genomes of their respective wild-type strains were sequenced using long-read technology. These sequences were combined with the short-read sequences obtained previously [1] to generate closed and complete genome assemblies to use as a reference. By comparing the genomes of cured and wild-type clones, we were able to confirm that only plasmid pOXA-48 was eliminated during plasmid curing (Table S5).

To test if our system was able to generate near-isogenic plasmid-carrying and plasmid-free clones, we analysed genomic variants in the cured strains compared to the wild-type strains (Table S6). Only two strains, CF13c1 and K163c1, did not accumulate any mutations at all. The remaining seven strains presented between one and three SNPs. Additionally, two of the strains, J57c1 and K153c2, showed possible genomic rearrangements. For a detailed description of the mutations see Supplemental Results.

Next, we investigated if the observed SNPs could be caused by off-target activity of the CRISPR-Cas9 system. We compared the sequences of the sgRNAs used to cure pOXA-48 with the mutated regions and found that sgRNAs aligned poorly (see Supplemental Results for more details). Thus, it is unlikely that the mutations are the result of off-target effects of the curing system, and they probably accumulated randomly due to single cell bottlenecks during the curing process. In summary, despite accumulating a few mutations, we show that wild-type and cured strains are isogenic or near-isogenic. We therefore conclude that the CRISPR-Cas9 system can be reliably used to cure pOXA-48 from clinical enterobacterial isolates.

### Determination of pOXA-48 fitness effects

**Fig. 3.**
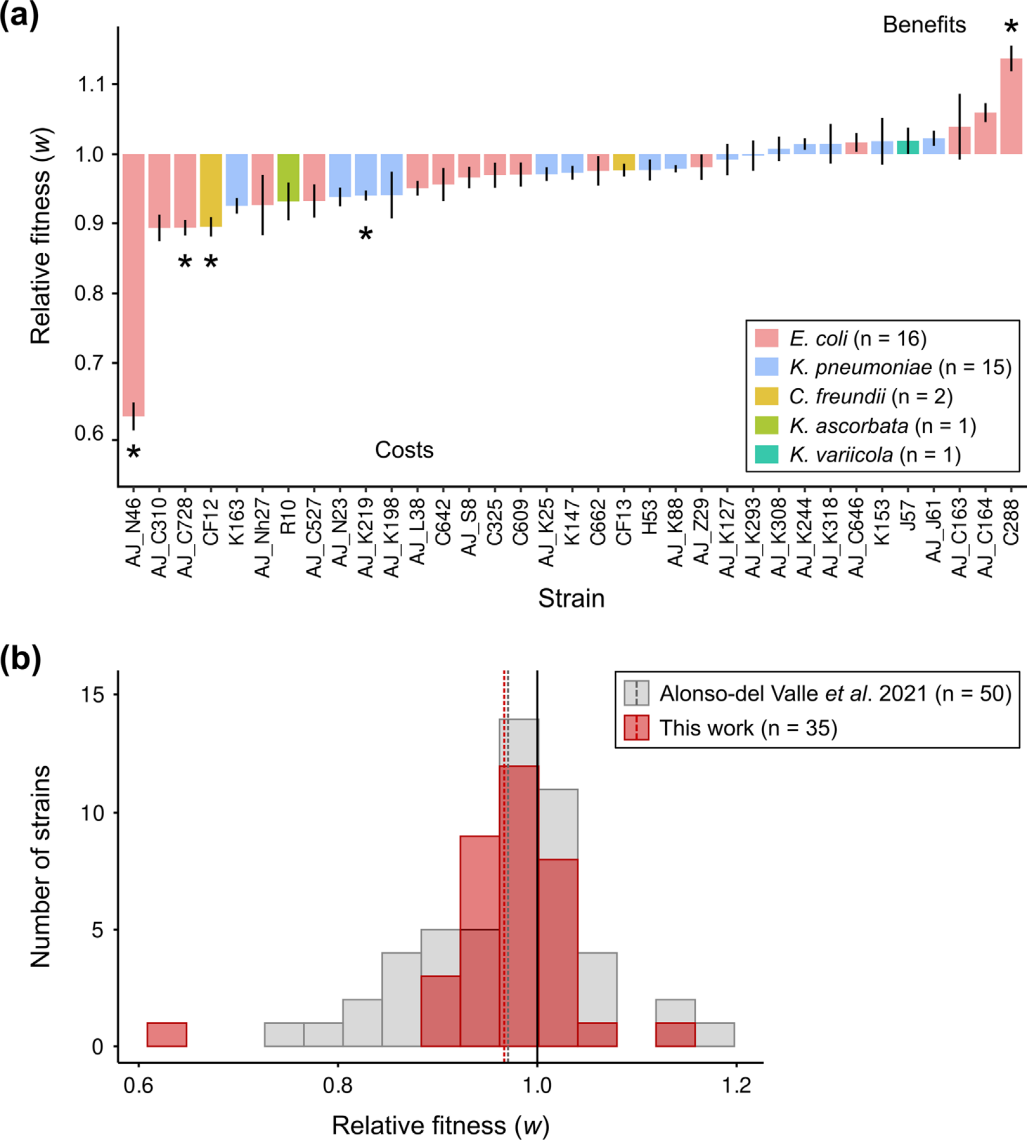
Fitness effects of plasmid pOXA-48 in wild-type clinical enterobacteria. (**a**) Relative fitness (*w*) of pOXA-48-carrying clones compared to their corresponding pOXA-48-free isogenic versions (see Methods). Values >1 indicate that plasmid pOXA-48 is associated with a fitness advantage, while values <1 indicate a fitness reduction associated with pOXA-48 carriage. Bars represent normalized relative fitness (average of six independent biological replicates of the competition pOXA-48-carrying vs. *E. coli* J53 divided by the average of six independent biological replicates of the competition pOXA-48-free vs. *E. coli* J53). Error bars represent the propagated standard error. Asterisks indicate significant costs or benefits associated with pOXA-48 carriage (Bonferroni-corrected paired *t* tests, *P*<0.05). Strain AJ_N46 is an outlier (Grubbs test, *P*=1.07×10^−6^). (**b**) Comparison of pOXA-48 DFE in the collection of naive, ecologically compatible pOXA-48 carriers [17] and in the collection of pOXA-48 natural carriers analysed in this work. Bars indicate the number of strains in each relative fitness bin. Dashed lines indicate the mean relative fitness of each collection. Both distributions are normal when not including the outlier AJ_N46 (Shapiro–Wilk normality tests: [17], *P*=0.14; this work, *P*=0.095). Cumulative distribution functions for both collections are represented in Fig. S4.

We performed competition assays to determine the fitness effects of plasmid pOXA-48 in the different strains of the collection (Fig. 3). We used flow cytometry to increase the throughput of these assays. Specifically, we competed each pOXA-48-carrying and pOXA-48-free clone against a fluorescently labelled *E. coli* J53 strain (*n*=70). We previously showed that this strain can be used as a common competitor against wild-type enterobacteria, producing comparable results to those from competitions between isogenic clones [17]. Moreover, as a control for the competition assays, we also performed individual growth curves of every clone in the collection (*n*=70). As expected, we observed a positive correlation between the relative fitness of the different clones measured in the competition assays against *E. coli* J53 and the area under the growth curves (AUC). AUC compiles different growth curve parameters [17, 39] (lag time, OD_max_ and *V*_max_) and can be used as a proxy of bacterial fitness (Spearman’s rank correlation, *rho*=0.626, *S*=20446, *P*=1.84×10^−8^; Fig. S2). Finally, we designed the competition assays to avoid the conjugation of plasmid pOXA-48 between the competitors, and we experimentally tested that neither conjugation nor plasmid loss affected the assay results (see Methods and Fig. S3).

We used the data from the competition assays to calculate the relative fitness of each wild-type pOXA-48-carrying clone compared to its pOXA-48-free isogenic version (Fig. 3a). The results showed that pOXA-48 produced a range of fitness effects, spanning from costs to benefits. Specifically, plasmid carriage was associated with a significant fitness cost in four strains and produced a significant fitness advantage in one strain (Bonferroni-corrected paired *t* tests, *P*<0.05). In the remaining 30 strains, pOXA-48 produced no significant change in relative fitness. Finally, and in line with our previous results [17], we observed no differences in the distribution of pOXA-48-induced fitness effects between *E. coli* and *K. pneumoniae* strains (Wilcoxon rank sum test, *W*=94, *P*=0.32; Fig. S4).

### Distribution of pOXA-48 fitness effects

We previously determined the DFE of pOXA-48 in naive but ecologically compatible enterobacteria from the gut microbiota of hospitalized patients. The plasmid produced variable fitness effects with a small fitness reduction on average (mean *w*=0.971, *var*=0.0072; Fig. 3b). Here, we reported the DFE in enterobacterial strains naturally carrying pOXA-48 recovered from patients in the same hospital (mean *w*=0.967, *var*=0.006; Fig. 3b). Our data revealed that pOXA-48 produced similar fitness effects in enterobacterial strains that were recovered with or without the plasmid from the same cohort of hospitalized patients (Fig. S5, two-sample *t*-test, *t*=0.23, d.f.=77.67, *P*=0.83; exact two-sample Kolmogorov–Smirnov test, *D*=0.17, *P*=0.56). Interestingly, our studies revealed that pOXA-48-carriage is not associated with fitness costs in the majority of wild-type enterobacterial strains from the gut microbiota of hospitalized patients, and this claim holds true both for clones naturally carrying pOXA-48 (31/35) and for pOXA-48-free clones (36/50).

## Discussion

The DFE of mutations is a central concept in genetics and evolutionary biology, with implications ranging from population adaptation rates to complex human diseases [40]. The fitness effects of new spontaneous mutations in bacteria follow a heavy-tailed distribution dominated by quasi-neutral mutations with infrequent strongly deleterious mutations [41, 42]. Horizontally acquired genes can also impose a fitness cost in bacterial hosts [43, 44]. However, horizontal gene transfer in bacteria is frequently mediated by mobile genetic elements, such as plasmids, that carry multiple genes. Over the last few years, many studies have quantified plasmid-associated fitness effects [8, 11, 45–50]. Nevertheless, most of our understanding of plasmid fitness costs comes from analysing the effects of foreign plasmids artificially introduced into new bacterial hosts, and we still know very little about the effects of plasmids in their native bacterial hosts. The difficulty of selectively removing plasmids from wild-type bacteria is responsible for this important limitation. However, recent advances in genome editing have allowed us to overcome this problem. Utilizing a previously developed CRISPR-Cas9-based curing system [21], we are now able to remove the carbapenem resistance plasmid pOXA-48 from wild-type, multidrug-resistant, clinical enterobacterial strains. This system presents both high efficiency (we were able to successfully cure 35 out of 44 clones transformed with the curing vector) and high specificity (no off-target effects were observed in the genomic analysis of the cured strains). Crucially, this curing system could be easily re-coded to remove virtually any plasmid from enterobacteria.

In this study, we determined the DFE of pOXA-48 in pOXA-48-carrying enterobacteria recovered from a cohort of hospitalized patients. Our findings reveal that the distribution of plasmid fitness effects in native hosts did not differ from that previously observed in ecologically compatible pOXA-48-free hosts from the same collection. The DFE was dominated by quasi-neutral mutations, with a slight shift towards fitness costs (Fig. 3b). Interestingly, this DFE produced by a mobile genetic element is similar to that of spontaneous mutations. It is important to mention that in our studies we divided the clinical isolates between pOXA-48-carrying or pOXA-48-free. However, it is impossible to rule out the possibility of the previous presence of this plasmid in the pOXA-48-free isolates. The similarity of pOXA-48 effects in both collections could therefore be the result of plasmid–bacterium preadaptations driven by the high mobility of pOXA-48-like plasmids in gut microbiota communities [1]. Alternatively, the DFE observed in the collection of pOXA-48-free wild-type strains could be explained by the high permissiveness to plasmid acquisition in this collection [46]. Regardless of the underlying mechanism, our results highlight the relevance of the ‘ecological compatibility’ between plasmids and their bacterial hosts. Supporting this idea, the costs observed in these two studies are lower than those observed in associations between plasmids and bacteria from different ecological origins (data form meta-analysis of 50 plasmid–bacterium pairs from 16 independent studies [45], mean *w*=0.91, *var*=0.029).

The results from this and other recent studies help to explain the high prevalence of pOXA-48-like plasmids in clinical environments [18]. First, pOXA-48 produces no or moderate fitness costs in most of the enterobacterial clones in the gut microbiota of hospitalized patients [17]. Second, pOXA-48 spreads through conjugation at high frequencies in these communities, allowing the plasmid to explore new bacterial hosts [1]. Third, pOXA-48–bacteria associations experience rapid within-patient evolution, promoting their adaptation to different antibiotic regimes [21]. We argue that the different eco-evolutionary dynamics that help resolve the ‘plasmid paradox’ for pOXA-48 will probably apply to many other plasmids across the wide diversity of natural microbiota.

## Supplementary Data

Supplementary material 1

Supplementary material 2

Supplementary material 3

Supplementary material 4

Supplementary material 5

Supplementary material 6

Supplementary material 7
