# Supplementary material for: The distribution of fitness effects of plasmid pOXA-48 in clinical enterobacteria
Source: Microbiology (Reading). 2023 Jul 28;169(7):001369. doi: 10.1099/mic.0.001369 (PMC10433420; doi:10.1099/mic.0.001369)
Supplement: Supplementary material 1 [file mic-169-1369-s001.pdf]

**Supplementary Material**

# The distribution of fitness effects of plasmid pOXA-48 in clinical enterobacteria

Ariadna Fernandez-Calvet[#,1], Laura Toribio-Celestino[#,1], Aida Alonso-del Valle[1], Jorge Sastre-Dominguez[1], Paula Valdes-Chiara[1], Alvaro San Millan[1,2,*], Javier DelaFuente[1,*]

1. Centro Nacional de Biotecnología (CNB), CSIC. Madrid, Spain.
2. Centro de Investigación Biológica en Red de Epidemiología y Salud Pública (CIBERESP), Instituto de Salud Carlos III, Madrid, Spain.

# Contributed equally

* Correspondence and request for materials should be addressed to Alvaro San Millan (asanmillan@cnb.csic.es) & Javier DelaFuente (fuentehidalgo91@gmail.com).

**Supplemental Results**

*Analysis of mutations affecting coding regions in cured clones*.

The genomes of the cured strains presented in total eight-point mutations in coding genes (Table S3). Four SNPs were predicted to have neutral effects on protein function: a synonymous variant in the iron donor protein CyaY in C288c2, a synonymous variant affecting the putrescine ABC transporter ATP-binding subunit PotG in C325c1, and two missense variants affecting a phage capsid protein in CF12c1 (SNAP2 score -88, accuracy 93%) and the type VI secretion system baseplate subunit TssF in H53c1 (SNAP2 score -64, accuracy 82%). It is worth noting that these mutations could indirectly impact protein function by altering gene expression[1,2]. The remaining four SNPs were predicted to alter protein function. The frameshift mutation affecting gene *pdeR* in CF12c1 compromises protein secondary structure (Figure S6), producing the elongation of the C-terminus (+22 amino acids), which comprises the EAL domain of PdeR. Mutations in this protein, a member of the curli fimbrae biosynthesis cascade, are related to increased biofilm formation[3]. Another mutation restored the reading frame of the frameshifted *znuB* gene in H53c1, a subunit of the zinc ABC transporter complex. The third variant introduced a premature stop codon that results in the loss of more than a third of an integrase domain-containing protein in strain K147c1. The loss of function of this protein would probably only affect integron biology, impeding its excision. Thus, it should not produce large-scale physiological changes in the cured strain with respect to the wild-type strain. The fourth SNP also introduced a premature stop codon in the *ompC* gene of C288c2. This resulted in a truncated version of the protein, cutting down from 367 to 171 amino acids. The nucleotide sequences of the mutated genes were submitted to a BLASTn search against the NCBI database. In C288c2, the synonymous SNP in the *cyaY* gene was also found in other enterobacteria (e.g. CP077379.1). In the case of H53c1, SNPs present in both *znuB* (as seen earlier, Figure S7) and *tssF* were also present in other enterobacteria (e.g. CP043597.1 and CP081896.1, respectively), suggesting these are naturally-occurring variants. Lastly, we detected two probable

insertion sequence (IS) rearrangements. One of them was a possible event of an IS jump from the IncF plasmid of J57c1 to its chromosome, inserting into the *yhjQ* gene and probably producing a knock-out. This gene is involved in the biosynthesis of cellulose, and its disruption can reduce bacterial aggregation and biofilm formation[4]. The other one was observed in K153c2: an IS1 element from its chromosome was integrated within a coding region of a putative AAA family ATPase, which are involved in a wide variety of functions, potentially producing a knock-out.

*Analysis of mutations affecting intergenic regions in cured clones*.

We analysed if the observed intergenic SNPs could be located within putative promoters. In the case of CF12c1, the SNP located 50 bp upstream *glpA* did not apparently fall within predicted promoters in the sense strand that could control the expression of *glpA* and downstream genes (Figure S8A). Still, this mutation could affect other regulatory regions or the transcription of non-coding RNAs. In J57c1, the SNP located 94 bp upstream the catecholate siderophore receptor *fiu* gene is positioned within a predicted promoter in the sense strand (final score 2.72) and near another promoter with higher score (2.79) (Figure S8B). Therefore, the expression of the *fiu* gene and downstream genes could be altered. In the IncF plasmid of J57c1 there are multiple intergenic mutations between positions 90533-90554 bp that could be due to the possible excision and insertion of the IS element into the chromosome of J57c1. These SNPs fall near predicted promoters for the sense strand, and thus, could affect transcription of the ISNCY-like element ISKpn21 family transposase (Figure S8C). Nonetheless, these mutations had low read coverage and alignment quality (multiple contiguous SNPs), and thus could represent false positive calls. In K153c2, another IS1 element was observed integrated into an intergenic region between the *fyuA* gene and a hypothetical protein, 77 bp upstream the latter. As with mutations in coding regions, the sequences of the mutated intergenic regions were also submitted to a BLASTn search against the NCBI database. However, we could not find the observed mutations in other organisms.

*Analysis of possible off-target events*. We aligned the sequences of the single-guide RNAs (sgOXA48 and sgPemK) to the regions surrounding the identified SNPs (see Methods). To recognize and produce off-target cuts, the alignment of the sgRNA must have less than eight mismatches, short or no gaps, and more importantly, the Cas9 protospacer adjacent motif (PAM) sequence NGG or NAG at the 3' of the sgRNA[5]. Most alignments had gaps and/or more than seven mismatches and lacked the PAM sequence. Only two alignments had less than eight mismatches and presented the PAM sequence NGG, constituting possible off-target events (Figure S9). First, sgOXA48 aligned against the region encompassing the *znuB* mutation in H53 —that restores the reading frame of *znuB* in H53c1— with only five mismatches (Figure S9A). However, the mutation affecting *znuB* in H53c1 is a naturally-occurring variant. In fact, we could not find any enterobacteria in the NCBI database that carried the frameshift variant of H53. Second, the sgPemK guide aligned against the region surrounding the *ompC* mutation in C288 (Figure S9B). However, the combination of gaps and/or mismatches located within the PAM-proximal region of the sgRNAs would most probably impede Cas9 cleavage[6]. Moreover, none of the other analysed strains showed mutations in the *ompC* nor the *znuB* genes, contradicting the possibility of off-target CRISPR-Cas9 activity.

## Supplementary Figures

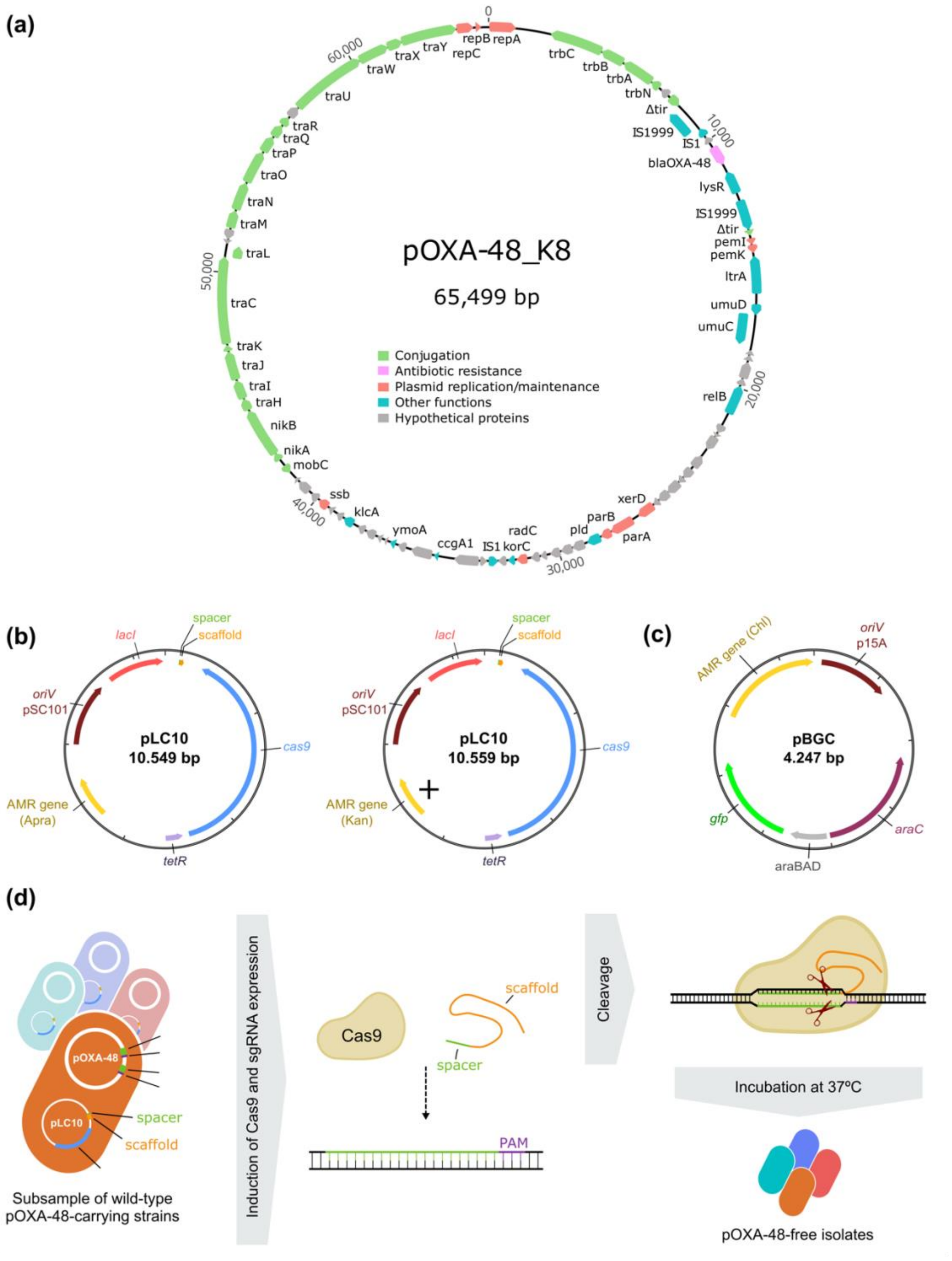


**Figure S1.** Plasmids used in this study and plasmid pOXA-48 curing protocol. (a) Genetic map of pOXA-48_K8 (accession number MT441554), the most prevalent plasmid variant in our collection. Open reading frames are represented with arrows.

Colours indicate gene functions (see legend). (b) Genetic maps of pLC10 plasmids, encoding the CRISPR-Cas9 system and a gene conferring resistance to apramycin (left) or kanamycin (right). The *cas9* gene and the single-guide RNA (sgRNA: spacer + scaffold) are under the control of the anhydrotetracycline (ATC)-inducible and isopropyl β-d-1-thiogalactopyranoside (IPTG)-inducible promoters $P_{tet}$ and $P_{lac}$, respectively. pLC10 also encodes a thermosensitive replication initiation protein (pSC101-based) that is not functional at 37ºC. (c) Genetic map of pBGC (accession number MT702881), used in competition assays. (d) Experimental design of pOXA-48 curing protocol (see Methods). Plasmid pLC10, encoding the sgRNA targeting either the $bla_{\text{OXA-48}}$ or *pemK* pOXA-48 genes (see PAM sequence 5'-NGG-3' in pOXA-48), was transformed into a subsample of wild-type pOXA-48-carrying enterobacterial strains. The expression of the *cas9* gene and the sgRNA was induced with ATC and IPTG, respectively, allowing cleavage of pOXA-48 at either target. Plasmid pLC10 was eliminated by incubating at 37ºC. pOXA-48-free strains were selected from colonies growing only in LB agar plates without antibiotics.

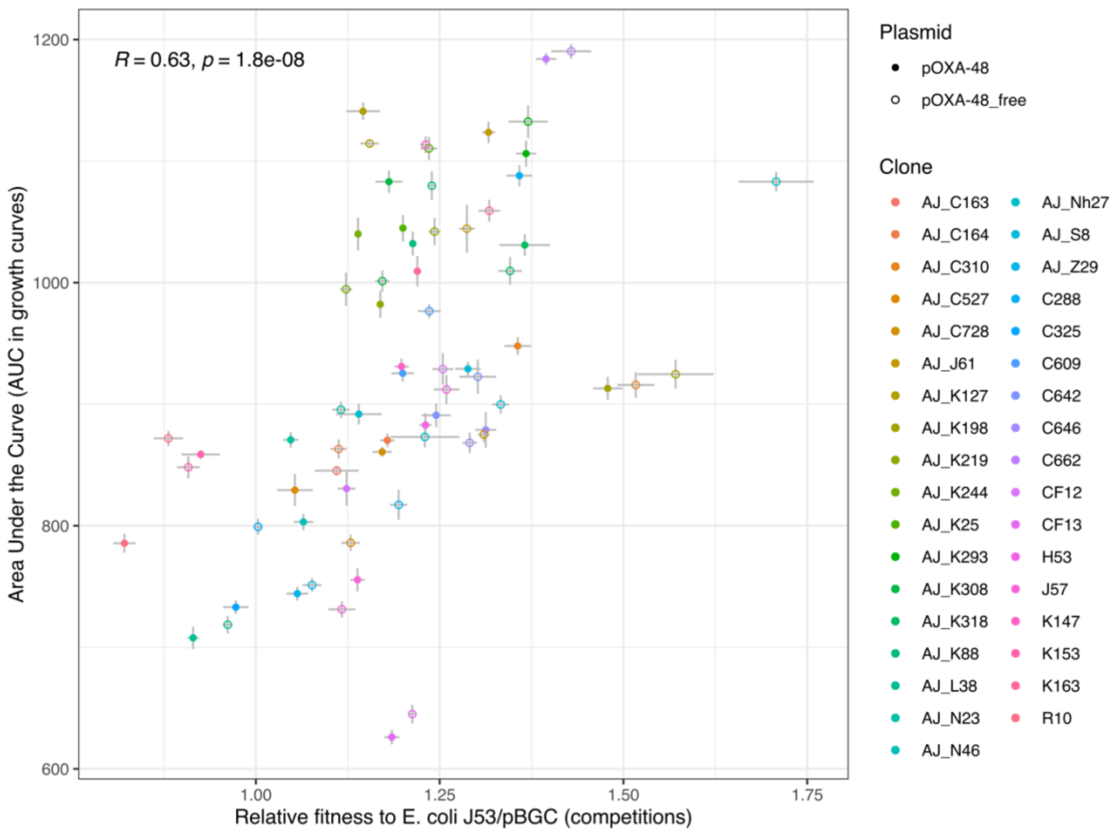


**Figure S2.** Correlation between the relative fitness and the area under the growth curves of each pOXA-48-carrying and pOXA-48-free clones. Note that the relative fitness of each clone was obtained by competing each pOXA-48-carrying and pOXA-48-free clone against a common competitor (*Escherichia coli* J53/pBGC). Each dot indicates the mean value (n = 6 for competition assays and n = 8 for growth curves). Lines indicate the standard error of the mean. Clones are indicated by colours and the presence/absence of pOXA-48 is indicated by full or empty circles. The Spearman's rank correlation between the fitness of each clone relative to *E. coli* J53/pBGC and the area under the growth curve is indicated in the top-left section of the figure (Spearman's rank correlation *rho* = 0.626, *S* = 20446, *P* = 1.84 x $10^{-8}$).

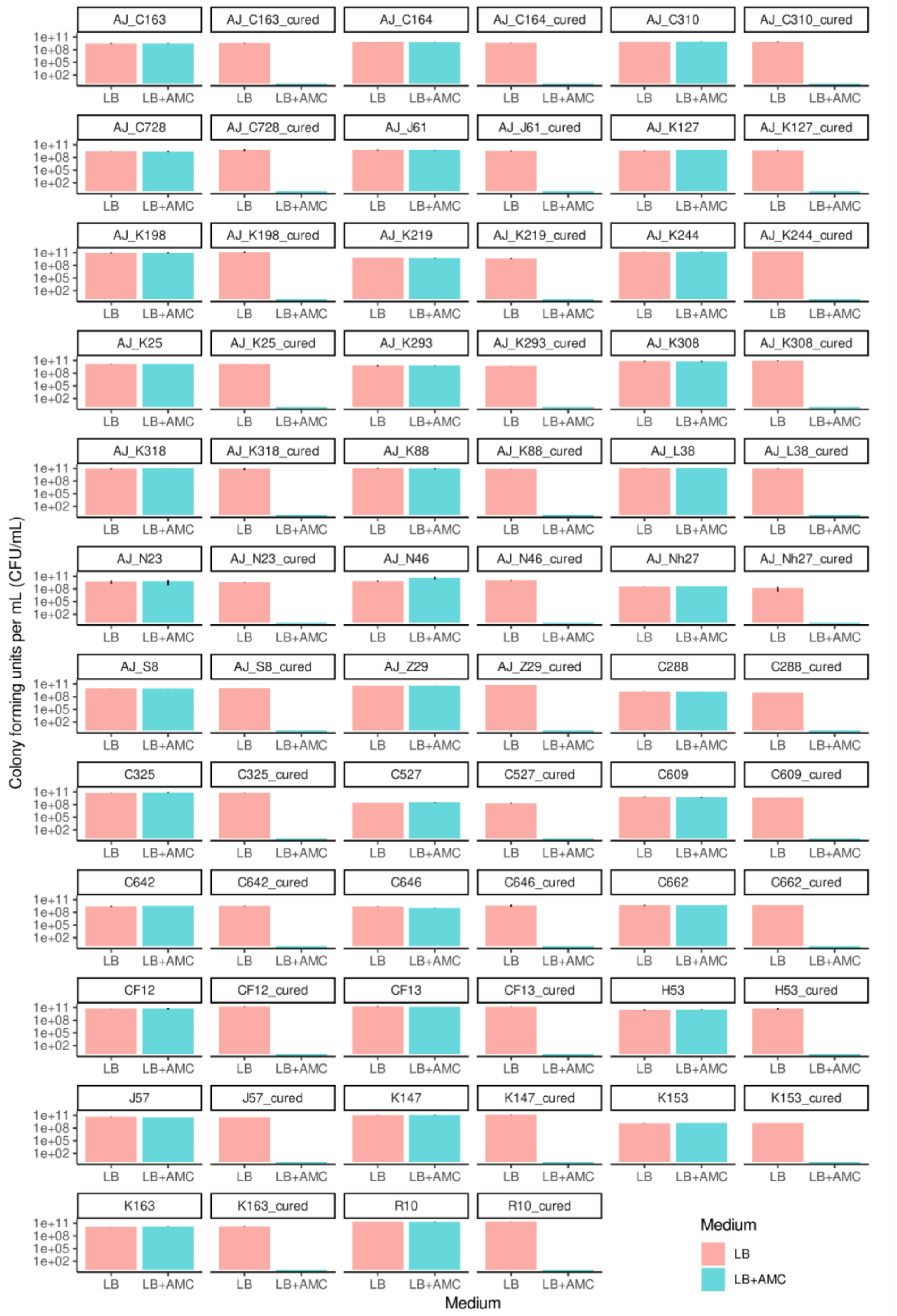


**Figure S3.** Measuring pOXA-48 loss during growth cycles. After growth curves, cells were serially-diluted and plated on agar plates supplemented with and without pOXA-48 selective antibiotics (AMC, Amoxicillin + clavulanic acid). Then, colony forming units (CFU/mL) were estimated (see Methods). Colours indicate each medium, bars indicate the CFU/mL and the error bars indicate the standard deviation of the mean. Note that (i)

in cured strains no colony forming units were detected in AMC treatments and (ii) there are no differences between CFU on selective and non-selective media for pOXA-48-carrying clones (Kruskal-Wallis, *chi-squared* = 0.00028, $P$ = 0.9867), indicating that plasmid pOXA-48 was maintained during the growth cycle.

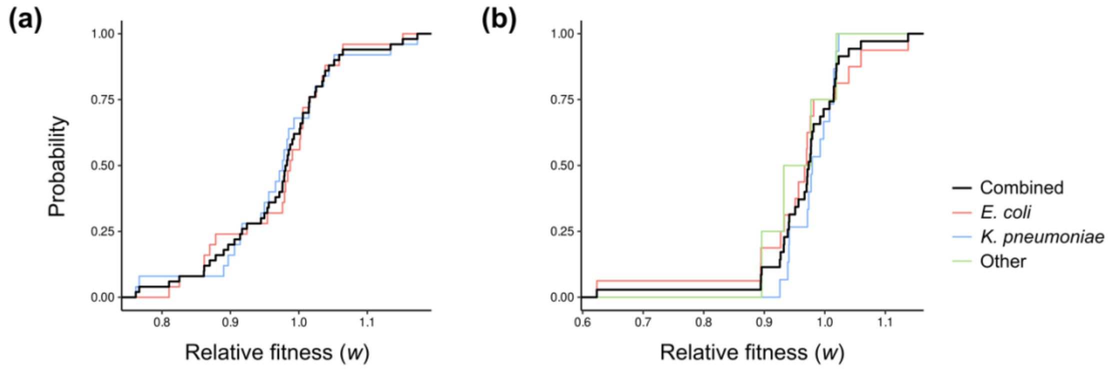


**Figure S4.** Cumulative distribution functions (CDF) of relative fitness (*w*) of pOXA-48-carrying strains. (a) CDF of the collection from Alonso-del Valle *et al*. 2021[7], which includes naive, ecologically compatible, pOXA-48-carrying enterobacteria (n = 50). (b) CDF of the collection of wild-type pOXA-48-carrying enterobacteria analysed in this work (n = 35). Lines indicate the CDF of all combined strains (black line) and strains separated by species (red line for *E. coli*, blue line for *K. pneumoniae* and green line for other species).

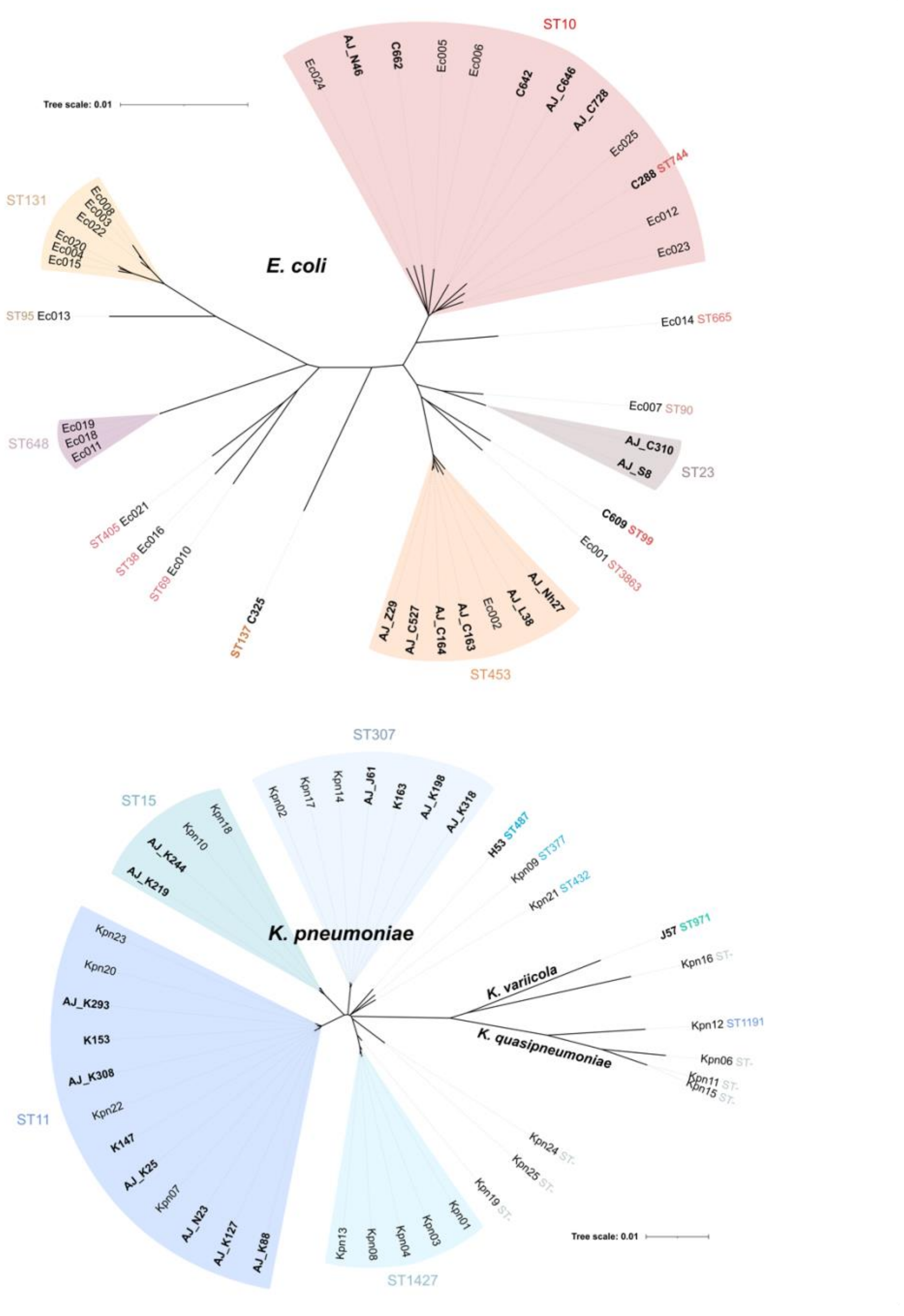


**Figure S5.** Unrooted phylogenetic trees of *Escherichia coli* (upper panel) and *Klebsiella* spp. (lower panel) strains from the collections of pOXA-48-cured wild-type enterobacteria used in this study and naive, ecologically compatible pOXA-48 carriers[7]. Phylogenies were constructed from mash distances between whole-genome assemblies, represented by branch lengths. Multilocus Sequence Types (ST) is indicated.

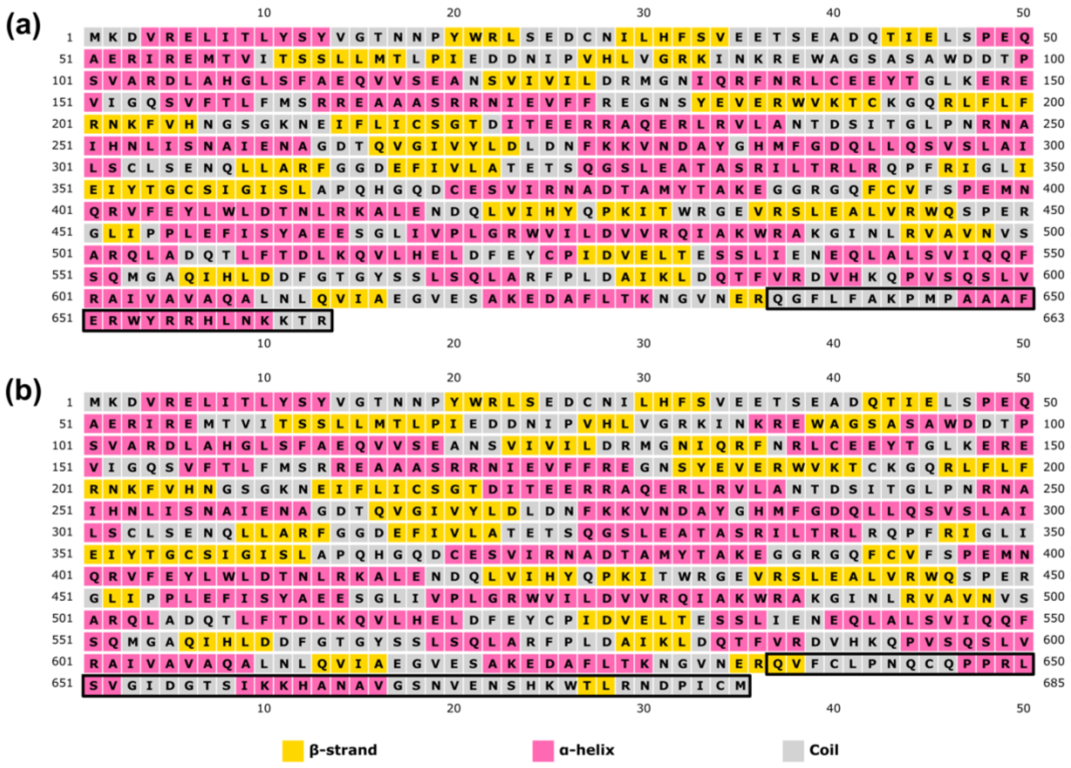


**Figure S6.** Secondary structure prediction by PSIPRED of PdeR. The PdeR protein of the wild-type CF12 strain has 663 amino acids (a). The frameshift variant of the cured strain affecting the residue Q638 of PdeR produces an elongation of +22 amino acids in the C-terminus that causes a change in secondary structure (b). The reference (a) and affected regions (b) are indicated by a black box.

| Score | Expect | Identities | Gaps | Strand |
|---|---|---|---|---|
| 1434 bits(776) | 0.0 | 783/786(99%) | 1/786(0%) | Plus/Plus |

```
Query  1    TCAGCTGGCGGCTTTTTTCGTCATGCTGAGAATAAACAGCACCGCGGCGCAGAGCACCAC  60
            ||||||||||||||||||||||||||||||||||||||||||||||||||||||||||||
Sbjct  1    TCAGCTGGCGGCTTTTTTCGTCATGCTGAGAATAAACAGCACCGCGGCGCAGAGCACCAC  60

Query  61   CGACGGCCCGGCCGGGGTATCATAGAAAGCCGAGAAGGTTAACCCGCCGGTAACGGCCAG  120
            ||||||||||||||||||||||||||||||||||||||||||||||||||||||||||||
Sbjct  61   CGACGGCCCGGCCGGGGTATCATAGAAAGCCGAGAAGGTTAACCCGCCGGTAACGGCCAG  120

Query  121  CATGCCCACGCCCACCGCCACGGCGGCCATCTGCTCCGGCGTACGGGCAAAACGGCGGGC  180
            ||||||||||||||||||||||||||||||||||||||||||||||||||||||||||||
Sbjct  121  CATGCCCACGCCCACCGCCACGGCGGCCATCTGCTCCGGCGTACGGGCAAAACGGCGGGC  180

Query  181  GGTGGCGGCCGGGATAATCAGCAGGGAAGTAATGATCAGCGCCCCGACGAACTTCATCGC  240
            ||||||||||||||||||||||||||||||||||||||||||||||||||||||||||||
Sbjct  181  GGTGGCGGCCGGGATAATCAGCAGGGAAGTAATGATCAGCGCCCCGACGAACTTCATCGC  240

Query  241  CACGCCGATAGTCAGGGCGGTGACCAGCATCAACAGCAGCTTGACCCGCTGCAGCTTCAC  300
            ||||||||||||||||||||||||||||||||||||||||||||||||||||||||||||
Sbjct  241  CACGCCGATAGTCAGGGCGGTGACCAGCATCAACAGCAGCTTGACCCGCTGCAGCTTCAC  300

Query  301  GCCGTCGACAAACGCAAGGTCCGGACTGATGGTCATCGCCAGCAGATTCCGCCACTGCCA  360
            ||||||||||||||| ||||||||||||||||||||||||||||||||||||||||||||
Sbjct  301  GCCGTCGACAAACGCCAGGTCCGGACTGATGGTCATCGCCAGCAGATTCCGCCACTGCCA  360

Query  361  GAGCAGAATGCCAATCACAATCACCACGCCAATGGCAATGGCGATCAGGTCCTGCGGCGT  420
            ||||||||||||||||||||||||||||||||||||||||||||||||||||||||||||
Sbjct  361  GAGCAGAATGCCAATCACAATCACCACGCCAATGGCAATGGCGATCAGGTCCTGCGGCGT  420

Query  421  AACCGCCAGCAGATCGCCGAACAGGTACGCCATCAGATCGACGCGGACGTTAGACATCAG  480
            ||||||||||||||||||||||||||||||||||||||||||||||||||||||||||||
Sbjct  421  AACCGCCAGCAGATCGCCGAACAGGTACGCCATCAGATCGACGCGGACGTTAGACATCAG  480

Query  481  ACTGACCACCACCAGCCCCAGGGACAGCGCGCTGTGCGCCATGATGCCCAGCAGGGTATC  540
            ||||||||||||||||||||||||||||||||||||||||||||||||||||||||||||
Sbjct  481  ACTGACCACCACCAGCCCCAGGGACAGCGCGCTGTGCGCCATGATGCCCAGCAGGGTATC  540

Query  541  GATTGCCAGATGCGGTCGTTTTTCCAGCCACACCAGCCCGCCAGCCAGCAGCAGGGTCAC  600
            ||| ||||||||||||||||||||||||||||||||||||||||||||||||||||||||
Sbjct  541  GATCGCCAGATGCGGTCGTTTTTCCAGCCACACCAGCCCGCCAGCCAGCAGCAGGGTCAC  600

Query  601  GGCGATCACCGCATAGAACGGATTGACGTTAAGCAATAAACCGAAAGCGACGCCGAGCAG  660
            ||||||||||||||||||||||||||||||||||||||||||||||||||||||||||||
Sbjct  601  GGCGATCACCGCATAGAACGGATTGACGTTAAGCAATAAACCGAAAGCGACGCCGAGCAG  660

Query  661  AGACGCATGCGCCAGCGTGTCGCC-AAATAAGACATGCGGCGCCAGACCACAAAGGAGCC  719
            |||||||||||||||||||||||| |||||||||||||||||||||||||||||||||||
Sbjct  661  AGACGCATGCGCCAGCGTGTCGCCGAAATAAGACATGCGGCGCCAGACCACAAAGGAGCC  720

Query  720  CAGCGGACCAGCGGCGCAGGCCAGCATCATCCCGGCAAGCCAGCCAGGTAACAGAAGTTC  779
            ||||||||||||||||||||||||||||||||||||||||||||||||||||||||||||
Sbjct  721  CAGCGGACCAGCGGCGCAGGCCAGCATCATCCCGGCAAGCCAGCCAGGTAACAGAAGTTC  780

Query  780  AATCAT  785
            ||||||
Sbjct  781  AATCAT  786
```

**Figure S7.** BLASTn alignment of H53's *znuB* (Query) and K147's *znuB* (Sbjct). The +G in K147 (also present in H53c1 and in other enterobacteria of the NCBI database), that restores the reading frame of *znuB*, is highlighted in orange.

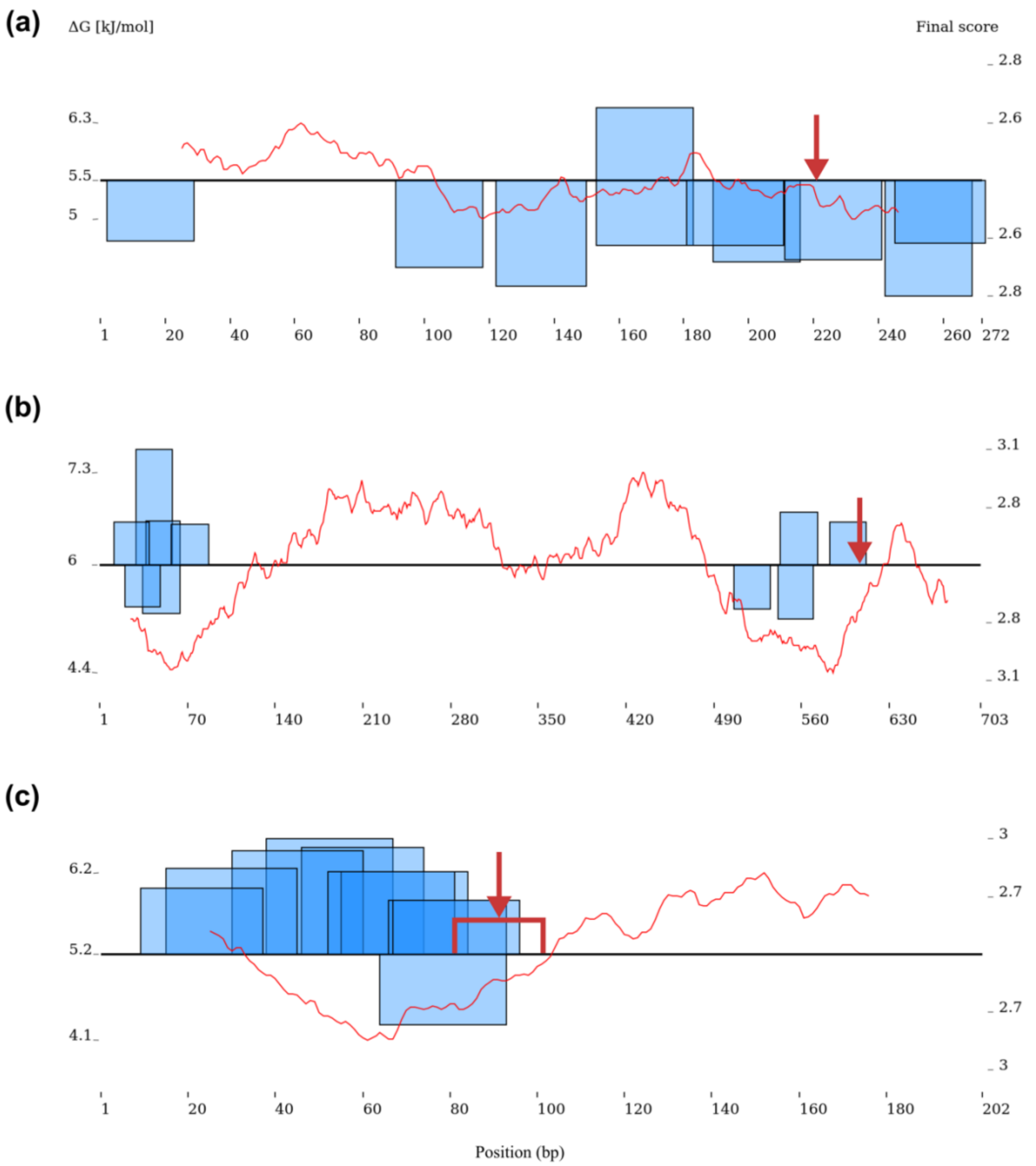


**Figure S8.** Putative promoters predicted by PromoterHunter in the mutated intergenic regions. (a) Intergenic region between genes *glpT* (antisense strand) and *glpA* (sense strand) in CF12. (b) Intergenic region between genes encoding the zinc-binding alcohol dehydrogenase family protein (sense) and the catecholate siderophore receptor Fiu (sense) in J57. (c) Intergenic regions between the genes encoding the GNAT family N-acetyltransferase (sense) and the ISNCY-like element ISKpn21 family transposase (sense) in the IncF plasmid of J57. In (a) and (b) the location of the SNPs is indicated with a red arrow. In (c), a red bracket also indicates the borders of the highly mutated region. Blue boxes above the horizontal black line represent putative promoters in the

sense strand; boxes below the horizontal line represent putative promoters in the antisense strand. Left *y* axis and the red line represent the Gibbs free energy ($\Delta G$) distribution along the sequence. Right *y* axis and the height of the blue boxes indicate the final scores of the putative promoters.

**(a)**

```
sgOXA48_fwd        1 ----------TGGCTTGTTTGACAATACGC-----------     20
                               ||.|||.||||.|.|.||||
H53_znuB_revc      1 TGGCGCCGCATGTCTTATTTGGCGACACGCTGGCGCATGCG     41
```

**(b)**

```
sgPemK_rev         1 ------CCGCTGGTCA-----CGGGCACAAC----------     20
                           ..||||.|||     .|||||.||.
C288_ompC          1 TGAACTTTGCTGTTCAGTACCAGGGCAAAAACGGTAGCGTA     41
```

**Figure S9.** Global alignments of sgRNAs against the regions enclosing mutations in H53's *znuB* (a) and C288's *ompC* (b). Only these alignments presented less than eight mismatches and the PAM sequence (NGG). The SNP position is indicated in red and the PAM sequence in purple. Mismatches are represented with dots. Fwd: forward sgRNA, rev: reverse sgRNA; revc: reverse complementary DNA sequence.

## Bibliography


1. Agashe, D. *et al.* Large-Effect Beneficial Synonymous Mutations Mediate Rapid and Parallel Adaptation in a Bacterium. *Mol Biol Evol* **33**, 1542–1553 (2016).
2. Lebeuf-Taylor, E., McCloskey, N., Bailey, S. F., Hinz, A. & Kassen, R. The distribution of fitness effects among synonymous mutations in a gene under directional selection. *Elife* **8**, e45952 (2019).
3. Weber, H., Pesavento, C., Possling, A., Tischendorf, G. & Hengge, R. Cyclic-di-GMP-mediated signalling within the σS network of Escherichia coli. *Mol Microbiol* **62**, 1014–1034 (2006).
4. Serra, D. O., Richter, A. M. & Hengge, R. Cellulose as an architectural element in spatially structured Escherichia coli biofilms. *J Bacteriol* **195**, 5540–5554 (2013).
5. Zischewski, J., Fischer, R. & Bortesi, L. Detection of on-target and off-target mutations generated by CRISPR/Cas9 and other sequence-specific nucleases. *Biotechnol Adv* **35**, 95–104 (2017).
6. Hsu, P. D. *et al.* DNA targeting specificity of RNA-guided Cas9 nucleases. *Nat Biotechnol* **31**, 827–832 (2013).
7. Alonso-del Valle, A. *et al.* Variability of plasmid fitness effects contributes to plasmid persistence in bacterial communities. *Nat Commun* **12**, 2653 (2021).
